# Promoting Recruitment using Information Management Efficiently (PRIME): a stepped-wedge, cluster randomised trial of a complex recruitment intervention embedded within the REstart or Stop Antithrombotics Randomised Trial

**DOI:** 10.1186/s13063-017-2355-z

**Published:** 2017-12-28

**Authors:** Amy E. Maxwell, Richard A. Parker, Jonathan Drever, Anthony Rudd, Martin S. Dennis, Christopher J. Weir, Rustam Al-Shahi Salman

**Affiliations:** 1 Centre for Clinical Brain Sciences, University of Edinburgh, Chancellor’s Building, 49 Little France Crescent, Edinburgh, EH16 4SB UK; 2 St Thomas’ Hospital, Westminster Bridge Road, London, UK; 3 Edinburgh Clinical Trials Unit and Centre for Population Health Sciences, Usher Institute of Population Health Sciences and Informatics, University of Edinburgh, Edinburgh, UK

**Keywords:** Methodology, Recruitment, Study within a trial, Stepped-wedge trial, Cluster randomised trial, Audit, Complex intervention

## Abstract

**Background:**

Few interventions are proven to increase recruitment in clinical trials. Recruitment to RESTART, a randomised controlled trial of secondary prevention after stroke due to intracerebral haemorrhage, has been slower than expected. Therefore, we sought to investigate an intervention to boost recruitment to RESTART.

**Methods/design:**

We conducted a stepped-wedge, cluster randomised trial of a complex intervention to increase recruitment, embedded within the RESTART trial. The primary objective was to investigate if the PRIME complex intervention (a recruitment co-ordinator who conducts a recruitment review, provides access to bespoke stroke audit data exports, and conducts a follow-up review after 6 months) increases the recruitment rate to RESTART. We included 72 hospital sites located in England, Wales, or Scotland that were active in RESTART in June 2015. All sites began in the control state and were allocated using block randomisation stratified by hospital location (Scotland versus England/Wales) to start the complex intervention in one of 12 different months. The primary outcome was the number of patients randomised into RESTART per month per site. We quantified the effect of the complex intervention on the primary outcome using a negative binomial, mixed model adjusting for site, December/January months, site location, and background time trends in recruitment rate.

**Results:**

We recruited and randomised 72 sites and recorded their monthly recruitment to RESTART over 24 months (March 2015 to February 2017 inclusive), providing 1728 site-months of observations for the primary analysis. The adjusted rate ratio for the number of patients randomised per month after allocation to the PRIME complex intervention versus control time before allocation to the PRIME complex intervention was 1.06 (95% confidence interval 0.55 to 2.03, *p* = 0.87). Although two thirds of respondents to the 6-month follow-up questionnaire agreed that the audit reports were useful, only six patients were reported to have been randomised using the audit reports. Respondents frequently reported resource and time pressures as being key barriers to running the audit reports.

**Conclusion:**

The PRIME complex intervention did not significantly improve the recruitment rate to RESTART. Further research is needed to establish if PRIME might be beneficial at an earlier stage in a prevention trial or for prevention dilemmas that arise more often in clinical practice.

**Electronic supplementary material:**

The online version of this article (doi:10.1186/s13063-017-2355-z) contains supplementary material, which is available to authorized users.

## Background

Under-recruitment to randomised controlled trials (RCTs) is a major source of inefficiency in the conduct of applied clinical research [1]. Slow recruitment delays the delivery of research and increases costs by increasing the number of staff and sites or by extending the amount and duration of funding required. If a trial extension is not possible, then under-recruitment will increase the likelihood of failing to detect a clinically relevant intervention effect if it exists, which may prevent patients from benefiting from a potentially efficacious intervention. This problem is extensive in clinical trials research – a review of 73 RCTs funded by the Medical Research Council (MRC) or Health Technology Assessment (HTA) in the UK in 2002–2008 found that almost half of the RCTs did not recruit their originally specified target sample size and nearly half of the RCTs received an extension of some kind [2].

The REstart or STop Antithrombotics Randomised Trial (RESTART, ISRCTN71907627, www.RESTARTtrial.org) is an on-going RCT comparing policies of restarting versus avoiding antiplatelet drugs for secondary prevention after stroke due to intracerebral haemorrhage (ICH), which aimed to recruit 720 participants over 2 years (from May 2013 to May 2015) based on recent epidemiological data [3, 4]. RESTART has been behind its recruitment targets despite implementing as many as possible of the strategies that have been shown to maximise recruitment [3, 5–7] including minimising the number of eligibility criteria and maximising the time window for recruiting patients after ICH onset.

The aim of the Promoting Recruitment using Information Management Efficiently (PRIME) trial was to investigate whether the rate of randomisation of patients to RESTART could be increased by means of a complex recruitment intervention applied to sites through which investigators in secondary care, with the support of a recruitment co-ordinator, used electronic patient records held by national stroke audits to identify potentially eligible patients.

## Methods/design

### Design and setting

**Fig. 1:**
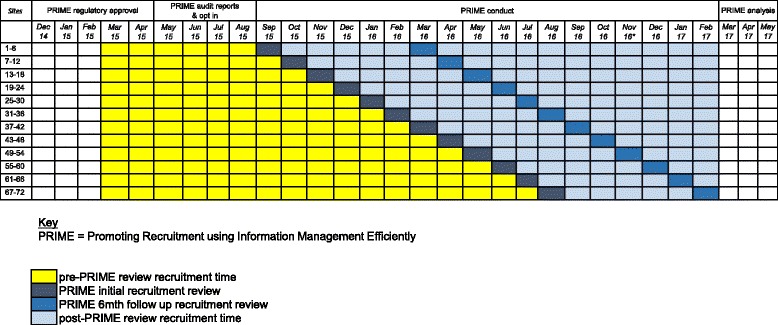
The Promoting Recruitment using Information Management Efficiently (PRIME) stepped-wedge trial design

PRIME is a closed-cohort, stepped-wedge, cluster randomised trial investigating a complex intervention to boost recruitment in RESTART. PRIME is an embedded recruitment trial within the RESTART parallel-group randomised trial. The stepped-wedge design involved a sequential roll out of the PRIME complex intervention to clusters (active hospital sites in RESTART) over twelve 1-month time periods [8]. The PRIME trial is an example of a closed-cohort, stepped-wedge trial because all sites at the start of the trial were expected to continue until the end of the study and no new sites were to be added [9]. All sites began in the control state (no intervention). The month in which the sites started the PRIME complex intervention was randomly allocated so that groups of sites began the intervention sequentially at equally spaced time intervals (steps) as shown in Fig. 1.

### Complex recruitment intervention

The PRIME complex intervention involved a recruitment co-ordinator discussing ways to improve recruitment to RESTART at each site via teleconference with the principal investigator and/or the local team, focussing on the provision of software for each site with instructions on how to extract from national stroke audit data lists of their own patients who were potentially eligible for RESTART. There was then a second teleconference to review progress 6 months later. We sent each site a questionnaire before the recruitment review and then a follow-up questionnaire before the 6-month follow-up review. Full details of the PRIME complex intervention, along with inclusion/exclusion criteria and the sample size calculation can be found in the PRIME protocol publication [10].

We created bespoke stroke audit data exports for PRIME in collaboration with the Scottish Stroke Care Audit (SSCA) and the Sentinel Stroke National Audit Programme (SSNAP) teams, to reflect the RESTART eligibility criteria as closely as possible. We provided sites with instructions on how to produce these exports once they had received their recruitment review. Further details can be found in the PRIME protocol [10].

Each audit database produced two bespoke RESTART audit reports (short and long) which could be run separately. The short reports for both were most likely to have suitable patients for RESTART whilst the long reports widened the criteria, and contained further potentially eligible patients. Once the reports were generated the site staff checked that the patient met all the eligibility criteria, using the patient’s medical records and/or via their general practitioner.

The 6-month follow-up questionnaire asked sites to collect information on the number of patients randomised directly from the audit reports.

### Site recruitment

**Fig. 2:**
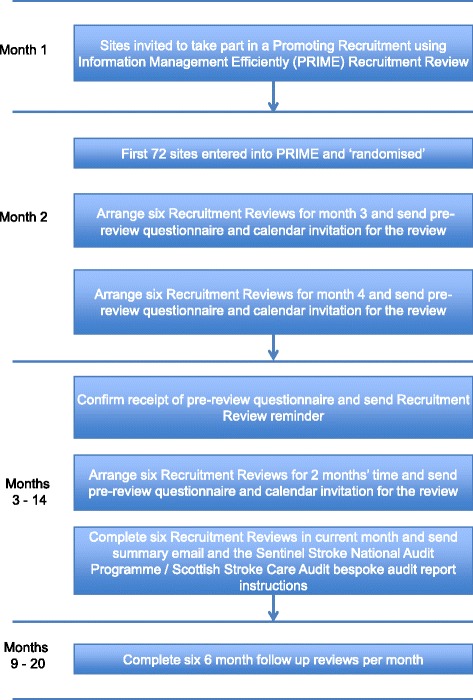
The Promoting Recruitment using Information Management Efficiently (PRIME) complex intervention flow chart

Active hospital sites in RESTART were invited to take part in PRIME in June 2015. There were 109 active hospital sites in the RESTART collaboration in the UK at that time. Twenty-four were excluded: two sites in Northern Ireland (where stroke audit data collection was not consistent); two sites in Scotland and two sites in England that piloted the bespoke stroke audit data exports; 14 sites recently activated to RESTART in 2015 (to ensure that all sites taking part had several months recruiting before receiving the intervention); and the remaining four excluded sites were unsuitable for other reasons. The PRIME recruitment co-ordinator telephoned the local RESTART co-ordinator at each of the remaining 85 sites in ascending order of site identification number to invite them to take part in a ‘recruitment review’, following up by email if required, until the sample size target of 72 sites agreed, as shown in the PRIME complex intervention flow chart (Fig. 2). Sites were not informed about the exact content of the recruitment review or that its timing would be randomly allocated. The recruitment co-ordinator and staff at each participating site remained blinded to the timing of the recruitment review until 2 months before the month allocated for each site’s review, when this had to be revealed in order to organise the review.

### Randomisation

We used stratified block randomisation to randomly allocate the 72 sites into 12 groups of six sites each, with stratification by hospital location (Scotland vs. England/Wales) to ensure that the proportions of sites with access to each national stroke audit data source were approximately consistent across the 12 groups. A programmer independent of the trial created the computer-generated random allocation sequence and sent this list to the trial data manager who then informed the recruitment co-ordinator at the required times for them to arrange the review.

**Fig. 3:**
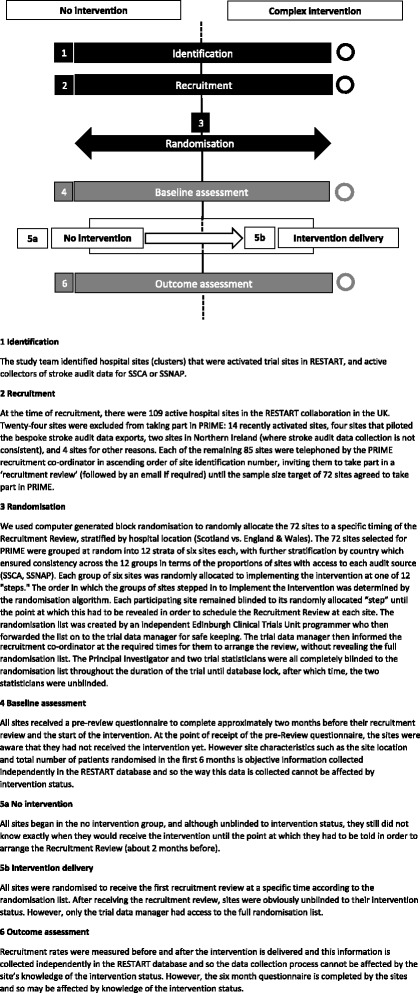
The Promoting Recruitment using Information Management Efficiently (PRIME) Timeline cluster diagram

A timeline cluster diagram is shown in Fig. 3 to clarify the timing of trial processes and blinding.

### Statistical methods

Full details of the statistical methodology can be found in the published Statistical Analysis Plan [11]. Briefly, the primary outcome of recruitment rate per site per month was analysed using a negative binomial generalised linear mixed model (GLMM), including the fixed-effect explanatory variables: number of months since start of the embedded PRIME study (represented by a linear term in the model to adjust for any background changes in trial recruitment rate), season (December/January versus all other months), site location (Scotland versus England/Wales), and an indicator variable for whether the complex intervention was due to have been implemented or not (according to the planned randomisation schedule). Site was also included as a random effect in the model.

The primary analysis followed an ‘as-randomised’ intention-to-treat principle, which meant that we analysed the data according to the allocated timing of the recruitment review, rather than the time that the recruitment review actually occurred. In addition, for the primary analysis, all 72 sites were included regardless of any site withdrawals or site suspensions or compliance with the PRIME trial procedures. For sites that were suspended (i.e. not permitted to recruit patients due to the principal investigator leaving or another reason), their missing observations were imputed as ‘0’ patients randomised. This was believed to be an appropriate imputation since it is likely, based on the reasons for suspension (e.g. due to the principal investigator being off sick), that many of these sites who were suspended would have failed to randomise any patients if they remained in the trial, and also ‘0’ was by far the most frequently observed number of patients randomised per month. However, to check the robustness of this assumption and the impact of any deviations from the protocol, a per-protocol secondary analysis was performed after excluding any sites experiencing permanent suspensions or sites failing to receive either the initial recruitment review or the 6-month follow-up review.

## Results

**Fig. 4:**
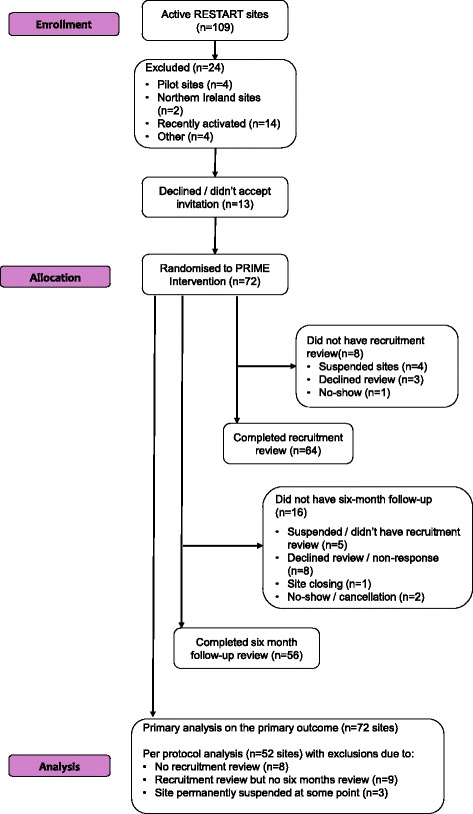
Consolidated Standards of Reporting Trials (CONSORT) 2010 flow diagram

The PRIME Consolidated Standards of Reporting Trials (CONSORT) flow diagram is shown in Fig. 4. Seventy-two sites were randomised to PRIME; of which 64 (89%) were located in England and Wales and eight (11%) in Scotland. Sixty-four sites (89%) received a recruitment review and 55 (76%) received both a first recruitment review and 6-month follow-up review.

### Recruitment review

The median delay between the scheduled randomised time of review (first day of the month allocated) and the actual time the recruitment review was delivered was 11.5 days (interquartile range (IQR) 8 to 19.75 days; range 0–71 days). In the 6-month period before any site received the PRIME complex intervention (March 2015 to August 2015), the total number of patients randomised was 0 for 47 sites (65%), 1 for 18 sites (25%), 2 for 6 sites (8%), and 3 for 1 site (1%).

Every time the recruitment review was conducted, we recorded whether there were any issues which impacted the PRIME complex intervention delivery. For 10 sites (14%), the sound quality for the teleconference was quite poor and for two sites (3%) it was very poor. This was due to a mixture of reasons including, sites using speaker phones and separate phones being used in the same room causing echo. For nine sites (12%) the principal investigator was absent from the review, and for three recruitment reviews (4%) the research co-ordinator was ill which may have affected delivery. For the 48 remaining sites (67%), we delivered the recruitment review with no issues.

Table 1 shows responses from the pre-recruitment review questionnaire. Eighteen sites (32%) were already using stroke audit data to recruit to RESTART; the most popular information source to identify eligible patients was screening logs (40 sites, 71%).

**Table 1:** Responses from the pre-recruitment review questionnaires

Approximate proportion of inpatients, who are suitable for follow-up, seen in clinic after hospital discharge	
0%	5 (12%)
10–30%	5 (12%)
60–90%	11 (26%)
100%	22 (51%)
Have you approached patients looked after by your stroke unit in the past to invite them back to clinic with a view to recruit them to RESTART?	
No	20 (37%)
Yes	34 (63%)
Have you used the template invitation letter to invite potential RESTART patients to clinic?	
No	37 (62%)
Yes	23 (38%)
Are your stroke audit data complete and accurate to the best of your knowledge?	
No	1 (2%)
Yes	57 (98%)
Are you already routinely using the stroke audit data to recruit to RESTART?	
No	39 (68%)
Yes	18 (32%)
What other sources of information do you have on patients which could be used to identify eligible RESTART patients? [tick all that apply]	
Screening logs	40 (71%)
A database other than the stroke audit	7 (12%)
Other	19 (34%)
No other information sources used (estimated based on number answering previous question but not this one)	6 (11%)
Total number of sites in at least one of above four categories	56
Have you used any other methods to boost recruitment?	
No	22 (41%)
Yes	32 (59%)
Have you found any barriers to finding suitable patients to recruit to RESTART?	
No	8 (15%)
Yes	46 (85%)

A complete summary of baseline site characteristics and responses to the pre-review questionnaire stratified by randomised group are shown in Additional file 1.

### Primary outcome

**Fig. 5:**
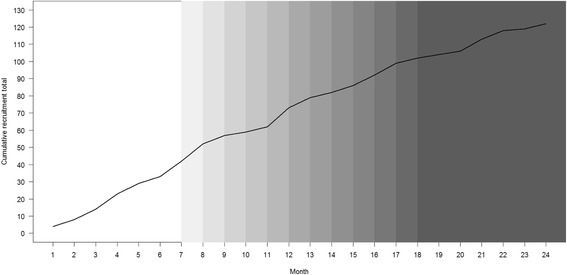
Change in cumulative randomisation total over time, with darker shading indicating more sites receiving the recruitment review

The change in the cumulative randomisation total (primary outcome) over time is shown in Fig. 5, with shading to indicate the number of sites that had actually received the recruitment review (i.e. started the intervention) up to that point. Separate plots for each of the randomised groups of sites are shown in Additional file 2.

Following imputation of zero monthly counts for nine suspended sites, each of the 72 sites contributed 24 months of observations (March 2015 to February 2017) and so the full primary analysis dataset constituted *N* = 1728 site-months of observations. Seventy-eight site-months (5%) were imputed with zero monthly counts due to site suspensions.

Table 2 shows the results of the primary analysis on the primary outcome. The adjusted rate ratio for the primary outcome (PRIME complex intervention versus control condition) was estimated to be 1.06 (95% confidence interval 0.55 to 2.03, *p* = 0.87) after adjusting for site, December/January months, site location (Scotland versus England and Wales), and time in months since the start of the embedded study.

**Table 2:** Negative-binomial generalised linear mixed model (GLMM) results for the primary analysis of the primary outcome (*N* = 1728, 72 sites)

Variable	Rate ratio	95% confidence interval	*p* value
Intervention (reference category: control condition)	1.06	0.55 to 2.03	0.870
Time since start of study (per month)	0.98	0.93 to 1.02	0.336
December/January (reference category: any other month)	0.55	0.288 to 1.05	0.071
Site location in Scotland (reference category: location in England or Wales)	2.00	0.96 to 4.2	0.063

### Per-protocol analysis

Nine sites were permanently suspended during the PRIME study period and, therefore, these sites were excluded from the per-protocol analysis, as were 11 other sites who, for whatever reason, did not receive either a first recruitment review or a 6-month review. After restricting the analysis to the per-protocol population of sites, the adjusted rate ratio became 1.01 (0.50 to 2.04, *p* = 0.98).

### Sensitivity analyses

The results were very similar and conclusions unchanged after performing other pre-specified sensitivity analyses (see Additional file 3). In particular, after restricting the analysis to the intervention roll-out period only (September 2015 to July 2016), the adjusted rate ratio became 1.07 (0.54 to 2.13, *p* = 0.84). Fitting a second-order spline function to model any secular changes over time improved the precision of estimation and reduced the width of the confidence interval slightly, but there was no change to the conclusions (RR 1.06, 95% CI 0.58 to 1.92, *p* = 0.86).

### Follow-up questionnaires

Fifty-one out of 72 sites (71%) returned the 6-month follow-up questionnaire, and 30 of these (59%) were known to have generated and used the audit data extract reports.

Out of the 30 sites using the audit reports, five sites (17%) ‘strongly agreed’ that the reports were useful, 15 sites ‘agreed’ that the reports were useful (50%), eight sites were ‘neutral’ (27%), and two sites ‘disagreed’ that the reports were useful.

Sixteen sites (53%) indicated that they either had a problem running or using the reports or listed disadvantages to using the reports. The most common disadvantage given was lack of time (seven sites, 23%); and a further three sites (10%) had problems with resource issues: for example, getting consultants or other staff to run the reports or review medical notes. Five sites (17%) complained that the procedure was not identifying many extra patients beyond those already screened as inpatients. Four sites (13%) specified that the reports were picking up a high number of ineligible patients and patients who had died. Two other sites (7%) were concerned about accuracy of the reports, indicating that ‘patients who are already in the trial are not in the list’, and ‘[some patients] weren’t a bleed at all’.

One site that used the audit reports made a suggestion for improvement: they wrote that they would have liked to have had ‘more information regarding understanding the report once produced, and how to link ID numbers to actual patients’.

Twenty-one sites answered ‘no’ to the question of whether they extracted and used the audit data extract reports. The most common barrier cited was resource/staffing/time issues (10/21, 48%); followed by use of other screening methods to identify RESTART patients (6/21, 29%); and problems running the reports or accessing the SSNAP database (6/21, 29%).

Where sites did create audit reports, these were run to an average of 3 years back (range 0.5–6 years) (see Table 3). Seventeen sites (68%) ran the audit reports once; seven sites ran the reports twice (28%); and one site ran the reports three times.

**Table 3:** Summary statistics for non-categorical responses to questions in the 6-month post-recruitment review questionnaire. Sites used short, long, or both types of bespoke RESTART audit reports

	Valid number of site responses	Median	Interquartile range (IQR)	Minimum	Maximum
How far back did you run the reports? (years)	28 (93%)	3.0	2.1 to 3.9	0.5	6
The number of patients identified by the audit reports					
Short	20 (67%)	17.5	8.75 to 84.50	3	273
Long	13 (43%)	26.0	16.5 to 96.0	2	787
Both	8 (27%)	45.5	16.25 to 236.25	10	376
The number of *eligible* patients identified by the audit reports					
Short	20 (67%)	1.0	0 to 4.0	0	25
Long	12 (40%)	0.5	0 to 2.5	0	15
Both	6 (20%)	4.5	0 to 8.75	0	11
The percentage of patients who were actually eligible out of all those identified by the audit reports					
Short	19 (63%)	4.0%	0 to 22.2%	0	100%
Long	12 (40%)	2.0%	0 to 9.8%	0	50%
Both	6 (20%)	4.5%	0 to 27.1%	0	50%
The number of eligible patients that the site contacted for sites identifying at least one eligible patient					
Short	12 (40%)	0.5	0 to 2.75	0	15
Long	5 (17%)	1.0	0 to 2.5	0	4
Both	5 (17%)	5.0	0 to 8.0	0	10
The percentage of patients who were actually contacted out of all those eligible for sites identifying at least one eligible patient					
Short	12 (40%)	25%	0 to 100%	0	100%
Long	5 (17%)	33.3%	0 to 100%	0	100%
Both	5 (17%)	62.5%	16.7% to 95.5%	0	100%
The number of eligible patients responding for sites contacting at least one patient					
Short	9 (30%)	0	0 to 1.0	0	15
Long	7 (23%)	0	0 to 0	0	2
Both	5 (17%)	1.0	0 to 7.0	0	10
The percentage of patients responding out of all those contacted for sites contacting at least one patient					
Short	9 (30%)	0%	0 to 75%	0	100%
Long	7 (23%)	0%	0 to 0%	0	50%
Both	5 (17%)	66.7%	0 to 100%	0	100%

Summary statistics for the number of patients identified by the reports, numbers invited back to clinic and numbers responding are shown in Table 3. Sites had the option to record their answers split by audit report (i.e. short and long), or if preferred, they had the option to record the combined total using the ‘Both’ category.

The number of eligible patients invited to a screening visit, declining to come to a screening visit, and numbers randomised is shown in Table 4.

**Table 4:** Number of patients reported to have come back to clinic and numbers randomised from the audit reports. Sites used short, long, or both types of bespoke RESTART audit reports

	The number of eligible patients who came to a screening visit				The number of eligible patients who *declined* to come to a screening visit				The number of patients who were randomised directly from the audit reports			
	0	1	2	3	0	1	2	≥3	0	1	2	3
Short	8	2	0	0	11	0	0	0	8	2	0	0
Long	6	1	0	0	6	1	0	0	6	0	0	0
Both	3	0	1	1	2	2	0	1	4	1	0	1

Under the ‘Short’ report category, two sites each reported one patient coming back to clinic; both patients were randomised. Under the ‘Long’ report category, one site reported a single patient coming back to clinic, but the patient was not randomised by the time of the 6-month questionnaire. Under the ‘Both’ category, the site with two patients coming back to clinic randomised one of them, and the site with three patients coming back to clinic managed to randomise all three of them. Therefore, the total number of patients randomised was six (half of whom were randomised through a single site). Although it should be noted, for this particular site, the data collected on the 6-month questionnaire was not from the bespoke audit data reports – they extracted a list of all ICH patients from the national audit data.

## Discussion

We found insufficient evidence that a complex intervention involving a recruitment review and using audit reports to search electronic records had a substantial impact on increasing recruitment to the RESTART trial. The point estimate of the rate ratio was close to 1 in the primary analysis and in all sensitivity analyses, including the per-protocol analysis. The corresponding 95% confidence limits indicate that a large intervention effect of rate ratio 2 or above is unlikely. Although most respondents (67%) to the 6-month questionnaire agreed that the audit reports were useful, only six patients in total were reported to have been randomised from the audit reports.

Since a detailed protocol was used to ensure fidelity to the intervention; and since 89% of sites received at least the first recruitment review and over 75% of sites received both reviews, the lack of intervention effect was unlikely to be due to failure to implement the intervention successfully. Indeed, even after removing sites that did not follow the protocol, no significant intervention effect was found.

There may be several reasons for the lack of evidence of an intervention effect. First, many sites reported a lack of time and resources to sufficiently search the audit data and use the bespoke audit reports. These issues were also raised as disadvantages by those sites who ran the audit reports. Secondly, it could be that the complex intervention itself was simply not effective. Our secondary outcome data suggested that the median percentage of patients identified by the audit reports that were eligible was low: less than 5%. Also the complex intervention did not include any face-to-face contact with the site, which may have reduced its effectiveness. The Study Within a Trial-1 (SWAT-1) study provided evidence of the effectiveness of an intervention involving a face-to-face site visit combined with scheduled meeting on recruitment rates in a multi-centre randomised trial. However, the findings of this study should be interpreted with caution due to the relatively weak study design (it was a non-randomised before-and-after study), and hence the potential for confounding bias [12].

Besides targeting the local site teams involved in recruitment, interventions could also be devised to target potentially eligible patients directly. Interestingly, a text-message intervention to potentially eligible participants stating that there was only a limited number of places left to join the ‘txt2stop’ smoking cessation trial was found to be effective in increasing recruitment rates compared to a text message with no such scarcity message content [13]. More recently, a cross-factorial, embedded RCT design within the EQUIP trial was used to investigate the effect of an intervention involving advertising patient and public involvement to boost recruitment rates. However, the authors found that the intervention had no significant effect on recruitment rates [14].

### Strengths and limitations

A strength of our embedded recruitment trial is that it has a cluster randomised design. Stepped-wedge or parallel-group cluster randomised designs are preferable to alternative non-randomised designs, such as before-and-after studies, because they are less susceptible to confounding bias due to temporal trends [15]. In cases where the potential harms or burden due to an intervention are known to be low, then the stepped-wedge design should be considered because all clusters receive a potentially efficacious intervention, and the trial may be more efficient compared to a parallel-group cluster trial. For recruitment trials embedded in multi-centre trials, it may be expected that there is a high level of heterogeneity in recruitment rates between sites due to substantial differences in site size and, therefore, a stepped-wedge trial design may confer greater power.

A limitation of our study is that the PRIME complex intervention was only implemented by a single recruitment co-ordinator which may limit the generalisability of results to other trials and settings. Even so, a detailed protocol was followed, so it is unlikely that this was a major limitation. The most important factor limiting generalisability was the fact that PRIME was embedded within a single host trial. If the intervention was implemented in other trials and settings the results may have been different.

In contrast, the START programme [16] was designed to test two recruitment interventions consisting of (1) optimised patient information sheets and (2) multimedia approaches to encourage patient involvement in research, across multiple host trials, which has provided more generalisable results compared to confining a recruitment intervention to a single host trial. The START investigators showed that an optimised patient information sheet did not significantly improve recruitment or retention rates into the REFORM trial [17].

A second but less important limitation relates to the 6-month follow-up questionnaire results. There was a limited response rate from sites to the first page of the 6-month questionnaire asking for number of patients randomised from the audit reports, which suggests that there may have been randomisations that resulted from the audit reports that were not recorded. Moreover, some sites reported that they were still working through the lists at the time of the 6-month review. Therefore, the self-reported results of the numbers randomised from the audit reports may only represent partial information. It should also be noted that for at least a few sites, the numbers given were from other lists of patients (apart from the audit reports) and some sites accessed the audit data directly without using the bespoke audit reports, albeit this could still be regarded as an indirect consequence of the intervention.

### Implications for further research

Although our study showed no significant effect of the PRIME complex intervention, this does not necessarily mean that similar recruitment interventions involving searching electronic patient records will not be effective in other trials or settings. Indeed, in primary care, using electronic patient records has been shown to boost recruitment to ongoing RCTs [18, 19]. Further work is needed to establish the effectiveness of using electronic patient records in secondary care. Alternative ways to increase recruitment may also need to be investigated, particularly those which minimise impact on clinician time and resources.

## Conclusions

We found insufficient evidence that the PRIME complex intervention is effective in increasing the recruitment rate to RESTART, despite the intervention being successfully implemented in the majority of sites. Further research is needed into methods to boost recruitment in trials in secondary care.

### Trial registration

PRIME was registered with the Northern Ireland Hub for Trials Methodology Research SWAT repository (SWAT22, http://bit.ly/2a4n7Yb) on 23 December 2015 and was submitted to the Online Resource for Recruitment research in Clinical trials (ORRCA, www.orrca.org.uk).

### Trial status

The host trial, RESTART, aimed to recruit 720 participants over 2 years, from May 2013 to May 2015 based on recent epidemiological data [3, 4]. Due to under-recruitment, RESTART’s funder granted an extension of recruitment until 31 May 2018. The trial end date is February 2019. PRIME’s first recruitment review took place in September 2015 and the last review completed in August 2016. The first 6-month follow-up review took place in March 2016 and the last one completed in February 2017. After the final 6-month follow-up was received there was a period of 3 months to analyse the data, meaning that PRIME completed in May 2017.

## Additional files

Additional file 1: Baseline site characteristics and responses to the pre-review questionnaire. (PDF 494 kb)

Additional file 2: Separate plots of each of the randomised groups of sites. (PDF 421 kb)

Additional file 3: Sensitivity analyses of the primary outcome. (PDF 278 kb)

## Abbreviations

CONSORT: Consolidated Standards of Reporting Trials
HTA: Health Technology Assessment
ICH: Intracerebral haemorrhage
MRC: Medical Research Council
NHS: National Health Service
PRIME: Promoting Recruitment using Information Management Efficiently
RCT: Randomised controlled trial
REC: Research Ethics Committee
RESTART: REstart or STop Antithrombotics Randomised Trial
SSCA: Scottish Stroke Care Audit
SSNAP: Sentinel Stoke National Audit Programme
SWAT: Study Within a Trial
