# Supplementary material for: Promoting Recruitment using Information Management Efficiently (PRIME): a stepped-wedge, cluster randomised trial of a complex recruitment intervention embedded within the REstart or Stop Antithrombotics Randomised Trial
Source: Trials. 2017 Dec 28;18:623. doi: 10.1186/s13063-017-2355-z (PMC5745698; doi:10.1186/s13063-017-2355-z)
Supplement: Supplementary file 1 — Baseline site characteristics and responses to the pre-review questionnaire. (PDF 494 kb) [file 13063_2017_2355_MOESM1_ESM.pdf]

Appendix 1 - Baseline site characteristics and responses to the pre-review questionnaire

**Month of PRIME intervention:**	**1**	**2**	**3**	**4**	**5**	**6**	**7**	**8**	**9**	**10**	**11**	**12**	**Overall**
Number of sites recruited and randomised	6	6	6	6	6	6	6	6	6	6	6	6	72
Number receiving the recruitment review intervention	6	5	6	6	5	5	5	5	5	5	6	5	64
Number receiving both the recruitment review intervention and follow-up review	6	4	6	5	5	4	4	3	5	3	5	5	55
Number of sites permanently suspended before end of PRIME follow-up period	0	1	1	1	0	1	2	1	1	0	1	0	9
Number receiving both interventions and active in RESTART throughout PRIME study period (i.e. included in per protocol analysis)	6	4	5	4	5	4	4	3	5	3	4	5	52
Number receiving both interventions, active in RESTART throughout PRIME study period and providing both pre-recruitment review and 6 months review questionnaire forms	6	4	3	3	3	1	4	3	5	3	4	5	44
Number of sites included in primary analysis	6	6	6	6	6	6	6	6	6	6	6	6	72

Appendix 1 - Baseline site characteristics and responses to the pre-review questionnaire

Month of PRIME intervention:	1	2	3	4	5	6	7	8	9	10	11	12	Overall
Site location													
England & Wales	5	5	5	5	5	5	5	5	6	6	6	6	64 (89%)
Scotland	1	1	1	1	1	1	1	1	0	0	0	0	8 (11%)
Time (days) between scheduled randomised time of review and actual time recruitment review was delivered													
N	6	5	6	6	5	5	5	5	5	5	6	5	64
Mean	15.2	6.8	14.5	21.5	16.6	12.6	11.6	12.4	12.4	15.8	16.5	17.2	14.6
Median	14.0	1.0	13.5	12.0	14.0	10.0	10.0	17.0	10.0	19.0	15.0	16.0	11.5
SD	10.6	11.5	6.7	24.5	5.50	8.7	8.7	7.7	5.4	9.8	8.3	7.3	10.6
Min	3	0	8	8	11	7	1	4	9	5	6	9	0
Max	29	27	24	71	25	28	21	19	22	27	27	29	71
1st Q	6	0.5	8.75	8.75	12.5	7.5	3.5	4.0	9.5	5.5	9.75	12.0	8.0
3rd Q	25	16	20.25	30.5	22.0	19.0	20.5	18.5	16.5	24.5	25.5	23.0	19.75
Any issues impacting intervention delivery													
No issues	4	2	3	6	3	6	4	4	2	4	5	5	48 (67%)
Recruitment co-ordinator had a bad cold	0	0	0	0	0	0	1	0	2	0	0	0	3 (4%)
No PI on the line	1	2	1	0	1	0	1	1	1	1	0	0	9 (12%)
Fairly poor sound quality	1	2	1	0	1	0	0	1	1	1	1	1	10 (14%)
Very poor sound quality	0	0	1	0	1	0	0	0	0	0	0	0	2 (3%)

Appendix 1 - Baseline site characteristics and responses to the pre-review questionnaire

**Month of PRIME intervention:**	**1**	**2**	**3**	**4**	**5**	**6**	**7**	**8**	**9**	**10**	**11**	**12**	**Overall**
Average number of patients randomised per month in 6 month period from March 15 to August 15													
0	3	5	2	4	3	4	4	3	4	6	5	4	47 (65%)
0.17 (1/6)	2	1	3	1	2	1	2	2	2	0	1	1	18 (25%)
0.33 (2/6)	1	0	1	1	0	1	0	1	0	0	0	1	6 (8%)
0.50 (3/6)	0	0	0	0	1	0	0	0	0	0	0	0	1 (1%)
Approx. proportion of In-Patients, who are suitable for follow-up, seen in clinic after hospital discharge (Q2 of Pre-recruitment review questionnaire)													
0%	0	2	1	0	0	0	0	2	0	0	0	0	5 (12%)
10-30%	2	0	0	1	0	1	0	0	0	1	0	0	5 (12%)
60-90%	0	1	4	0	1	0	0	0	0	3	1	1	11 (26%)
100%	2	0	0	2	2	2	4	2	3	0	3	2	22 (51%)

Appendix 1 - Baseline site characteristics and responses to the pre-review questionnaire

**Month of PRIME intervention:**	**1**	**2**	**3**	**4**	**5**	**6**	**7**	**8**	**9**	**10**	**11**	**12**	**Overall**
Have you approached patients looked after by your stroke unit in the past to invite them back to clinic with a view to recruit them to RESTART? (Q3 of Pre-recruitment review questionnaire)													
No	1	4	1	3	0	1	1	3	3	0	3	0	20 (37%)
Yes	5	1	4	1	2	2	4	1	2	4	3	5	34 (63%)
Have you used the template invitation letter to invite potential RESTART patients to clinic? (Q4 of Pre-recruitment review questionnaire)													
No	3	4	4	4	1	3	3	3	3	2	5	2	37 (62%)
Yes	3	1	2	0	3	1	2	2	2	3	1	3	23 (38%)

Appendix 1 - Baseline site characteristics and responses to the pre-review questionnaire

**Month of PRIME intervention:**	**1**	**2**	**3**	**4**	**5**	**6**	**7**	**8**	**9**	**10**	**11**	**12**	**Overall**
Are your stroke audit data complete and accurate to the best of your knowledge (Q5 of Pre-recruitment review questionnaire)													
No	0	0	0	0	0	0	0	0	1	0	0	0	1 (2%)
Yes	6	5	6	4	3	4	5	5	4	4	6	5	57 (98%)
Are you already routinely using the stroke audit data to recruit to RESTART? (Q7 of Pre-recruitment review questionnaire)													
No	4	3	2	3	2	3	5	2	4	3	5	3	39 (68%)
Yes	2	2	4	1	1	1	0	2	1	1	1	2	18 (32%)

Appendix 1 - Baseline site characteristics and responses to the pre-review questionnaire

Month of PRIME intervention:	1	2	3	4	5	6	7	8	9	10	11	12	Overall
What other sources of information do you have on patients which could be used to identify eligible RESTART patients? (Q8 of Pre-recruitment review questionnaire) [tick all that apply]													
Screening logs	3	5	3	3	4	4	2	3	3	3	4	3	40 (71%)
A database other than stroke audit	1	0	1	0	1	1	0	1	1	1	0	0	7 (12%)
Other	0	3	3	0	0	0	3	1	3	1	3	2	19 (34%)
No other information sources used (estimated based on number answering previous question but not this one)	2	0	0	1	0	0	1	0	0	0	1	1	6 (11%)
Total number of sites in at least one of above four categories	5	5	6	4	4	4	5	4	4	4	6	5	56 (100%)

Appendix 1 - Baseline site characteristics and responses to the pre-review questionnaire

**Month of PRIME intervention:**	**1**	**2**	**3**	**4**	**5**	**6**	**7**	**8**	**9**	**10**	**11**	**12**	**Overall**
Have you used any other methods to boost recruitment (Q9 of Pre-recruitment review questionnaire)?													
No	2	1	2	1	0	4	1	3	2	3	2	1	22 (41%)
Yes	4	4	3	3	2	0	4	1	2	1	4	4	32 (59%)
Have you found any barriers to finding suitable patients to recruit to RESTART? (Q10 of Pre-recruitment review questionnaire)													
No	1	1	0	0	0	2	0	2	0	2	0	0	8 (15%)
Yes	5	4	5	4	2	2	5	2	4	2	6	5	46 (85%)
