# Supplementary material for: Promoting Recruitment using Information Management Efficiently (PRIME): a stepped-wedge, cluster randomised trial of a complex recruitment intervention embedded within the REstart or Stop Antithrombotics Randomised Trial
Source: Trials. 2017 Dec 28;18:623. doi: 10.1186/s13063-017-2355-z (PMC5745698; doi:10.1186/s13063-017-2355-z)

Appendix 2: Separate plots of each of the randomised groups of sites

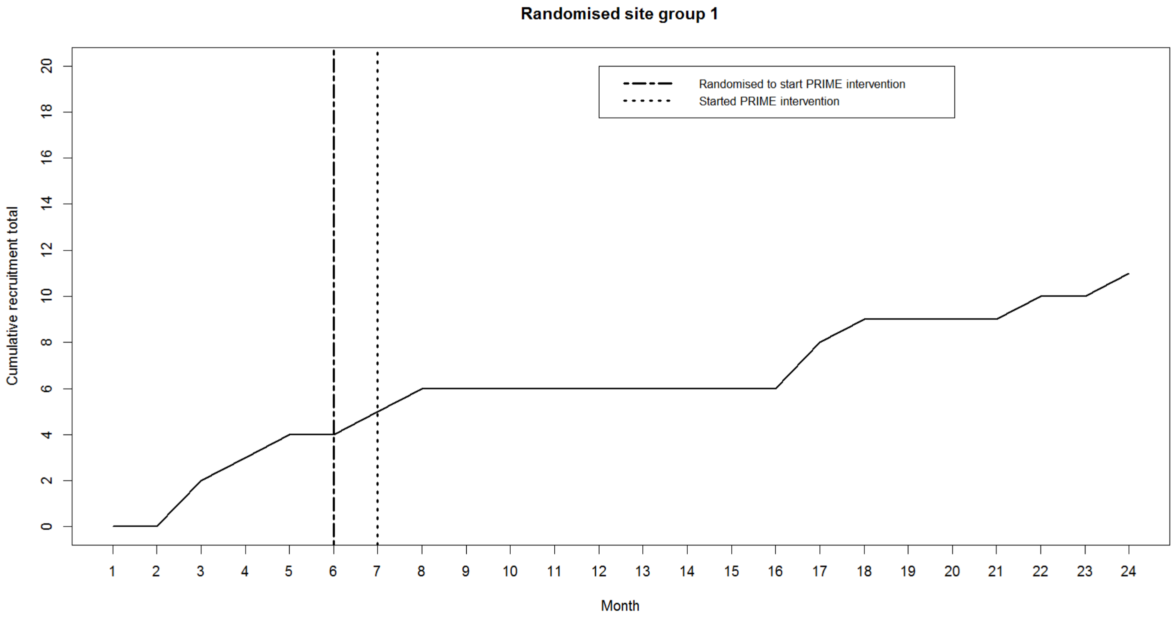

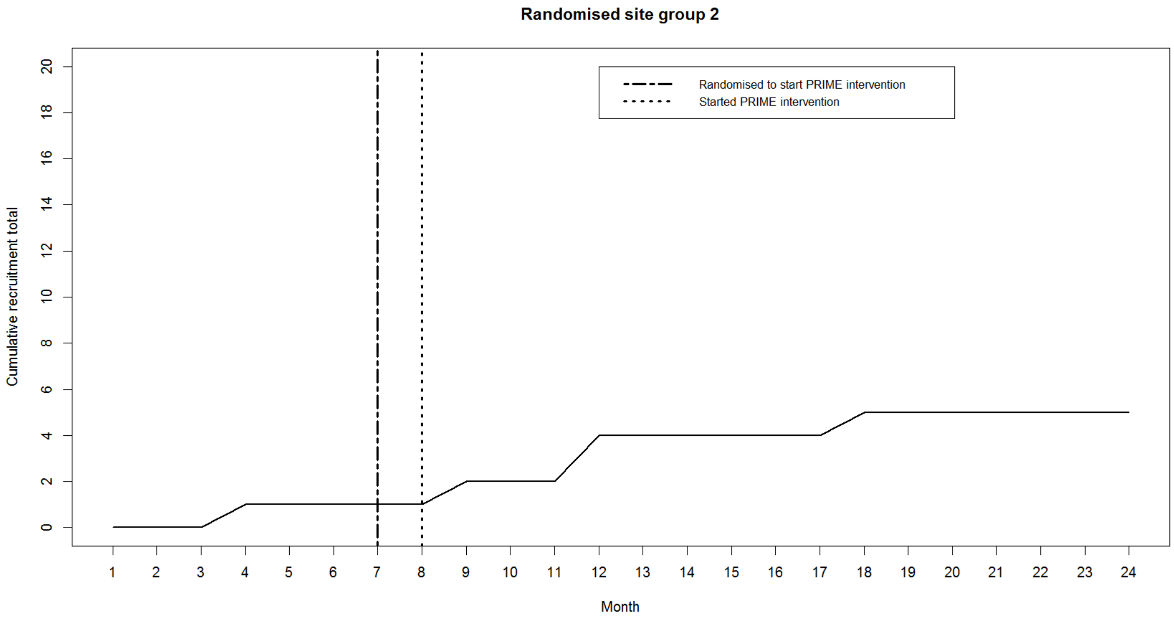

Appendix 2: Separate plots of each of the randomised groups of sites

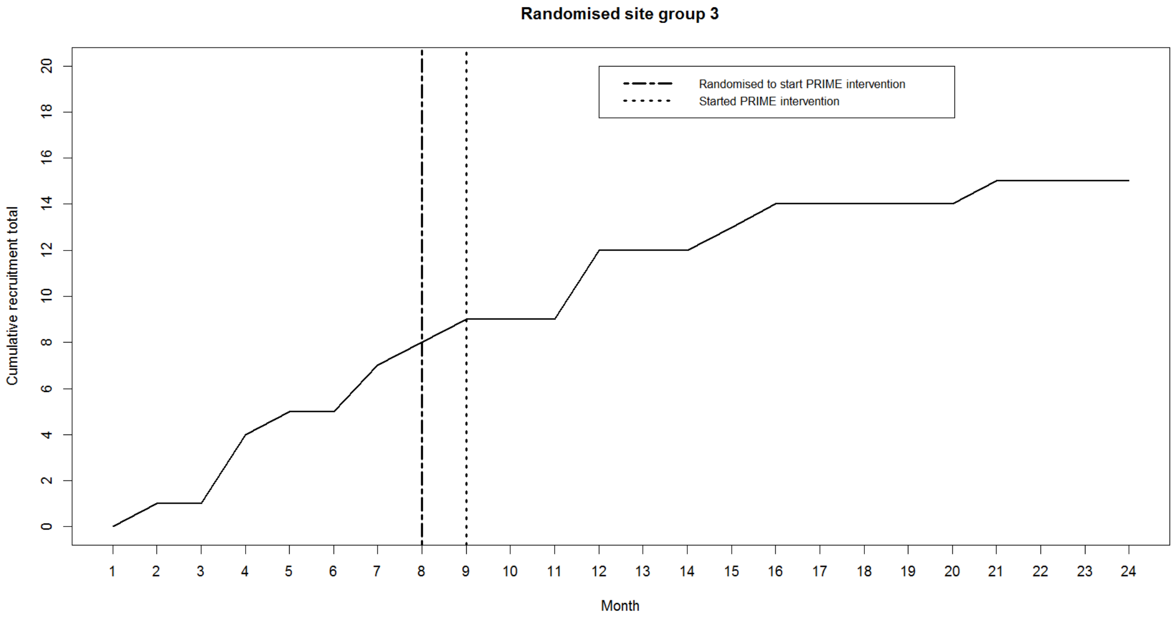

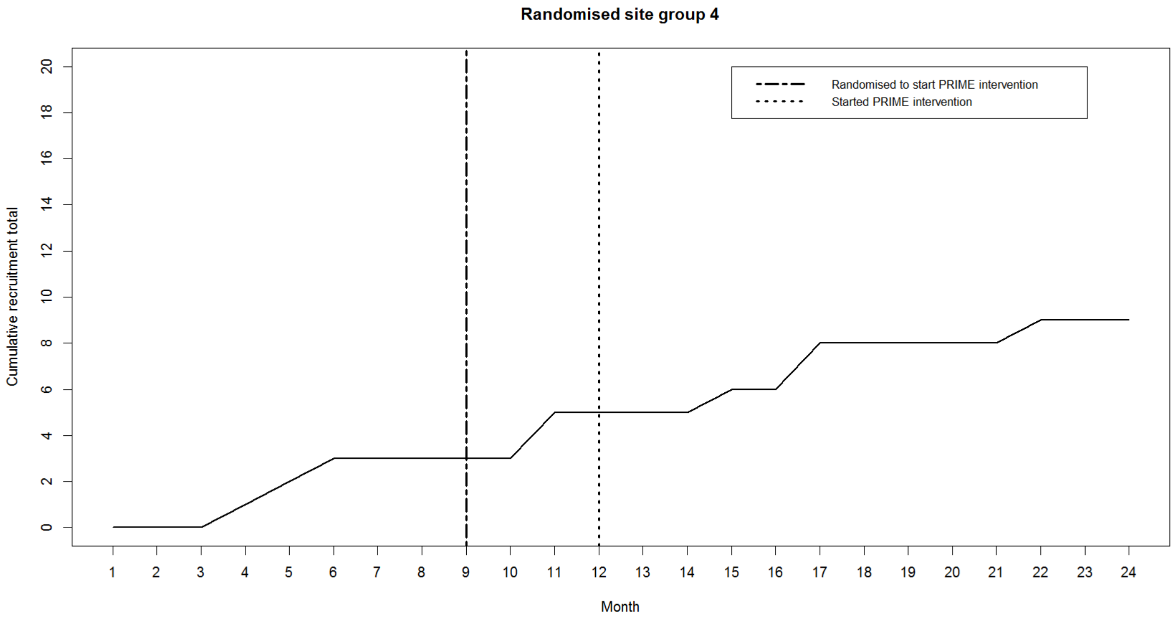

Appendix 2: Separate plots of each of the randomised groups of sites

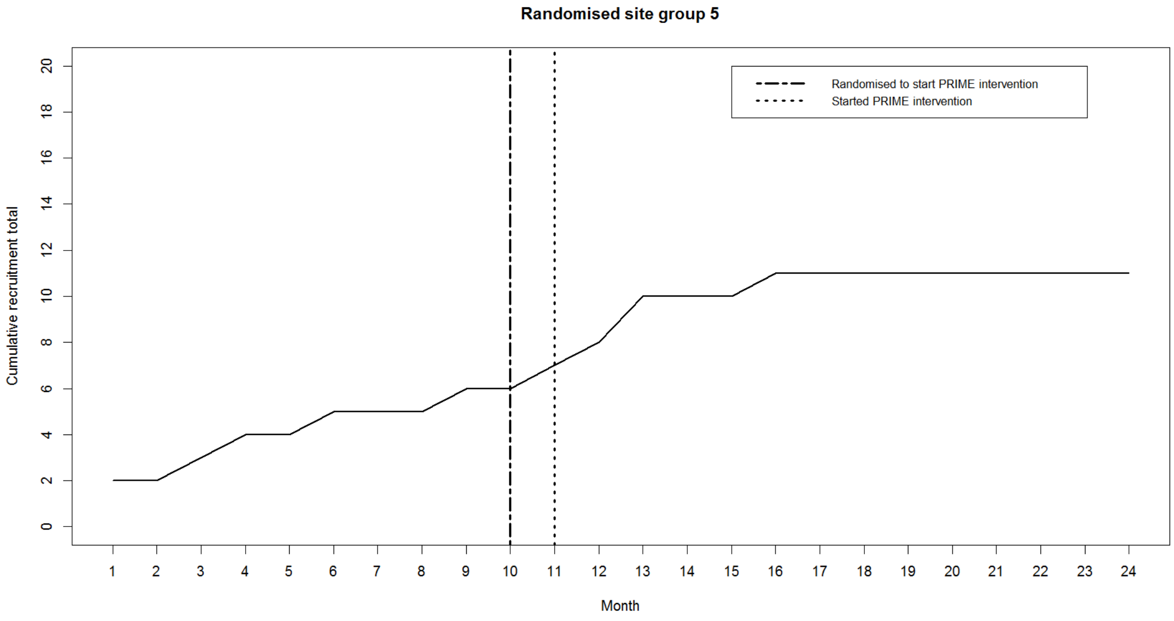

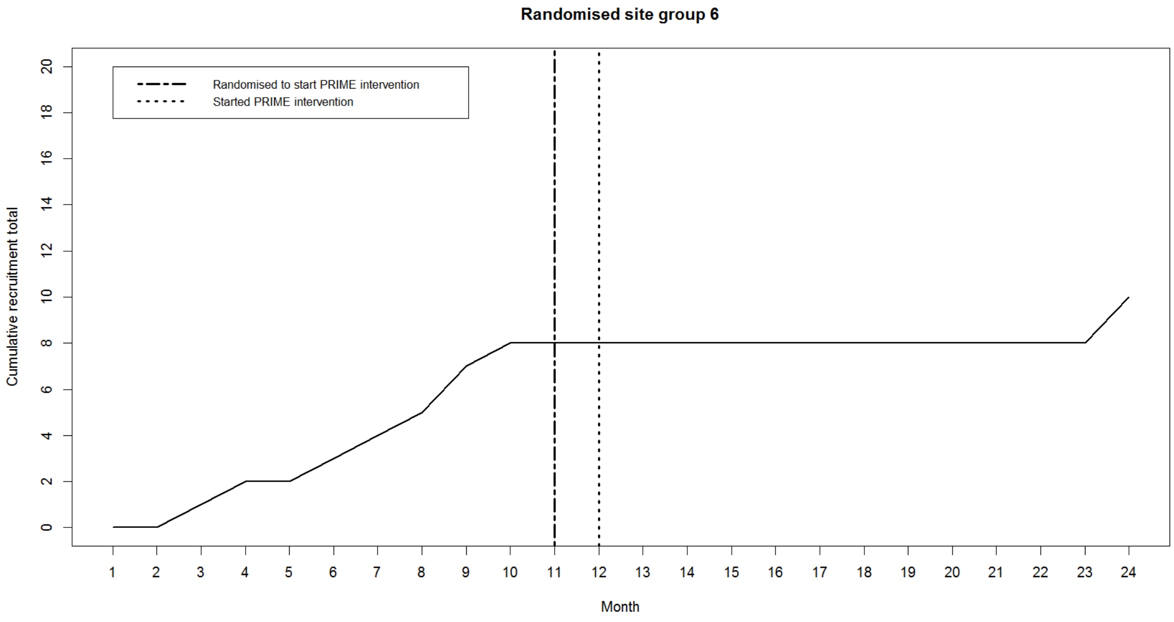

Appendix 2: Separate plots of each of the randomised groups of sites

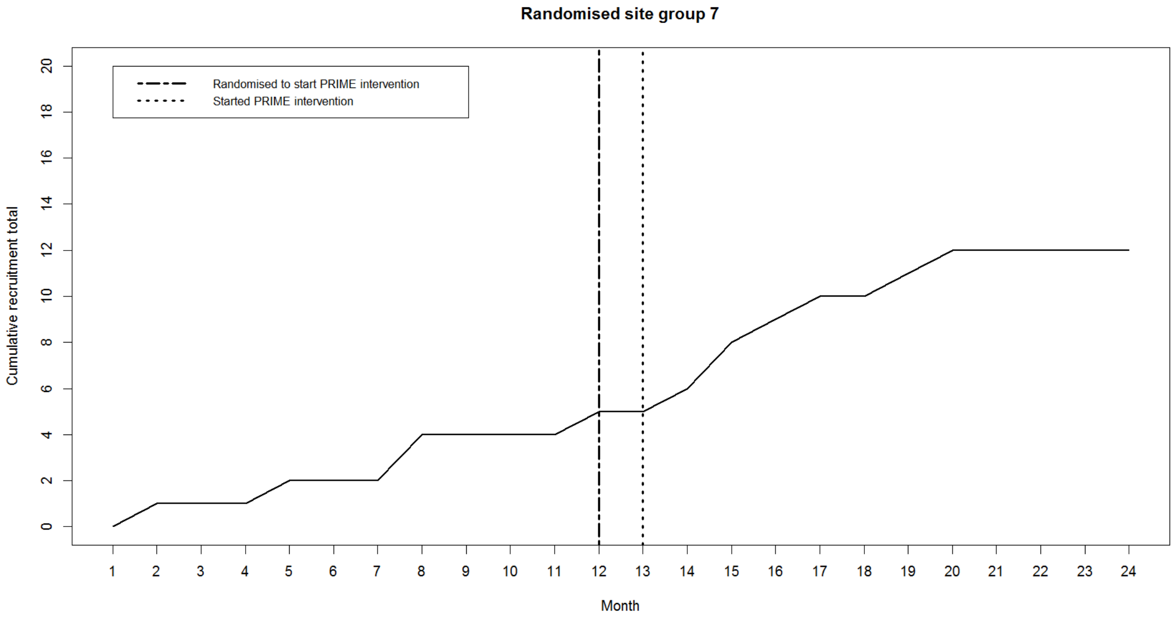

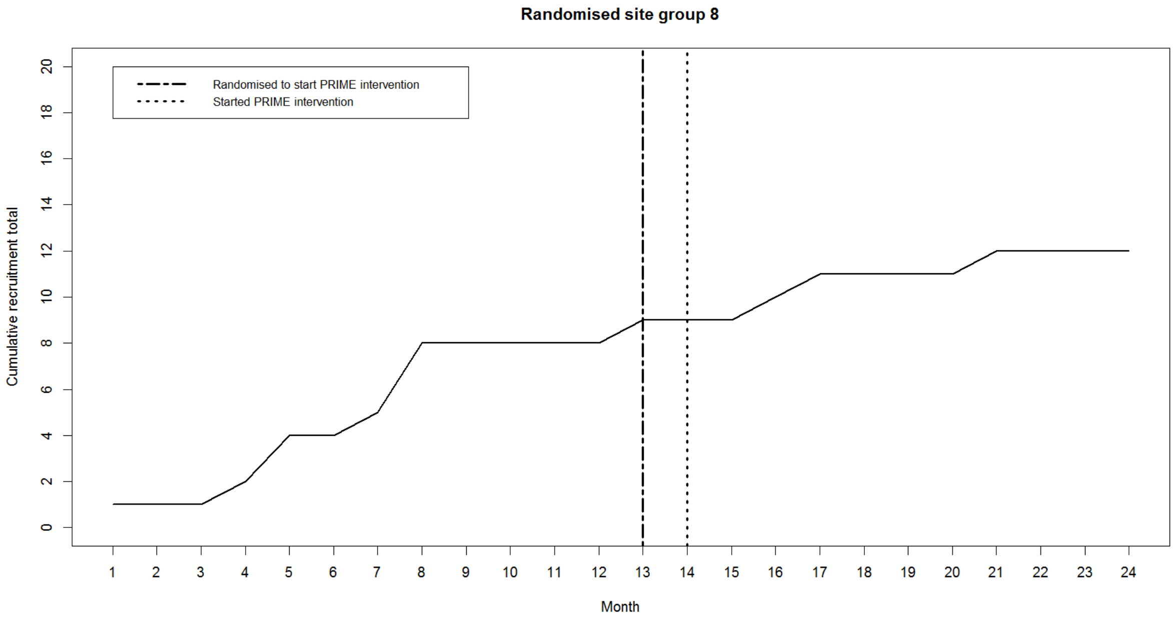

Appendix 2: Separate plots of each of the randomised groups of sites

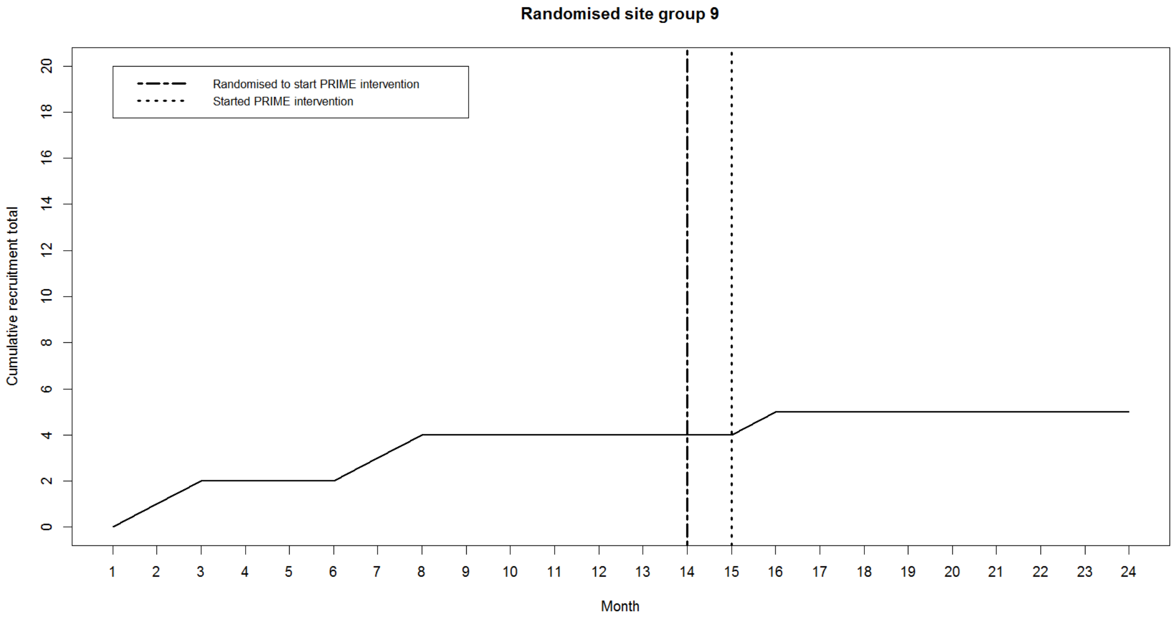

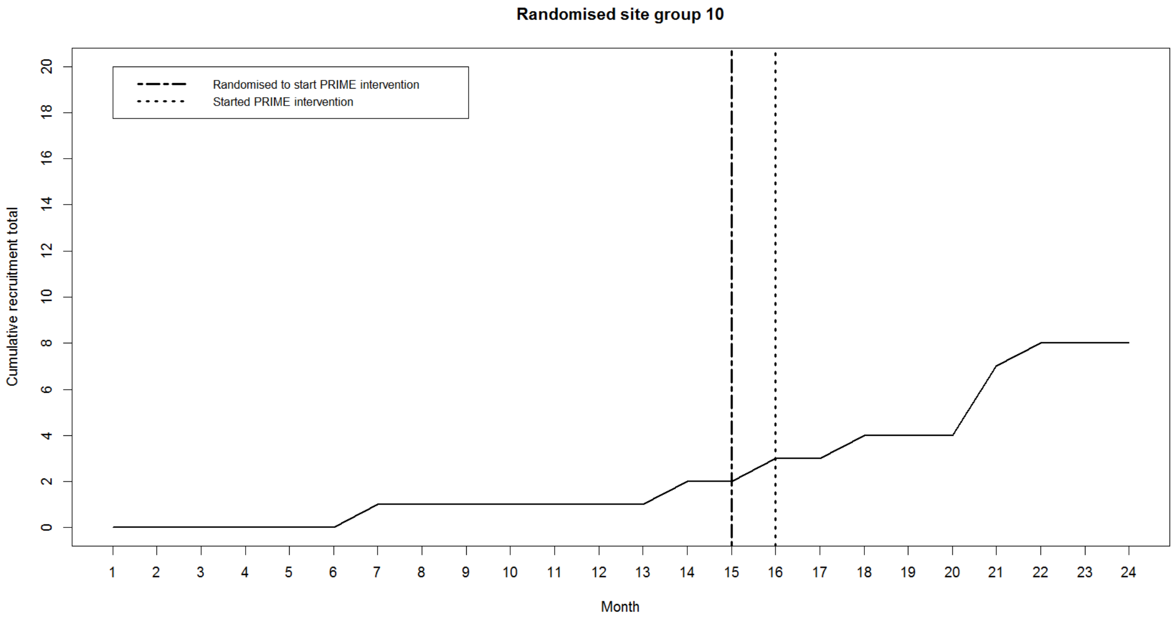

Appendix 2: Separate plots of each of the randomised groups of sites

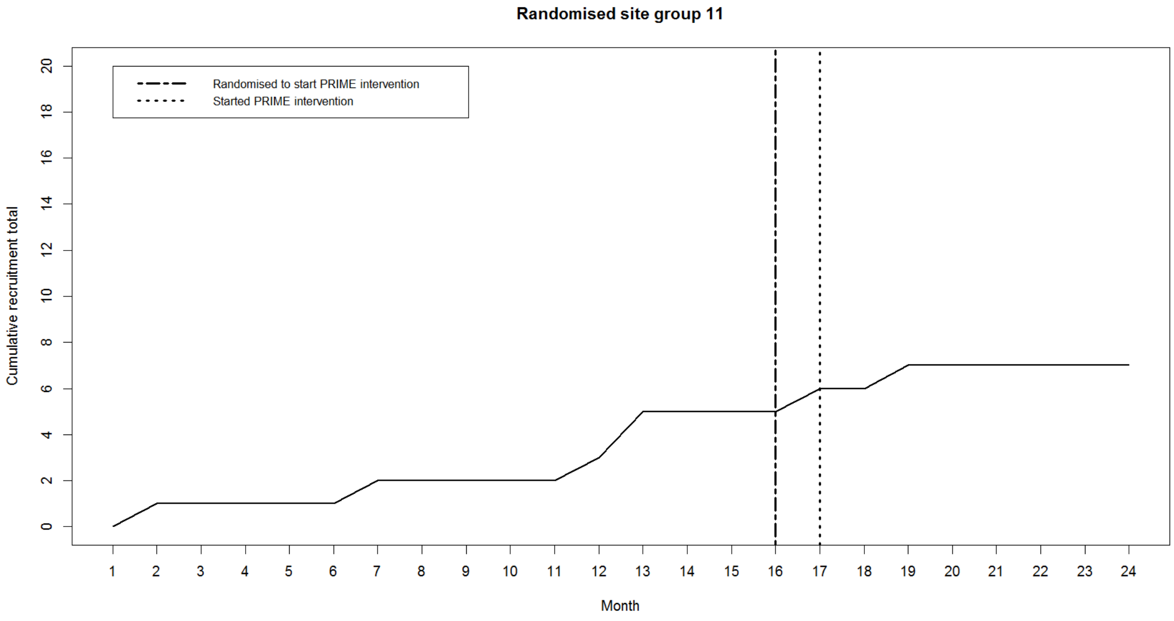

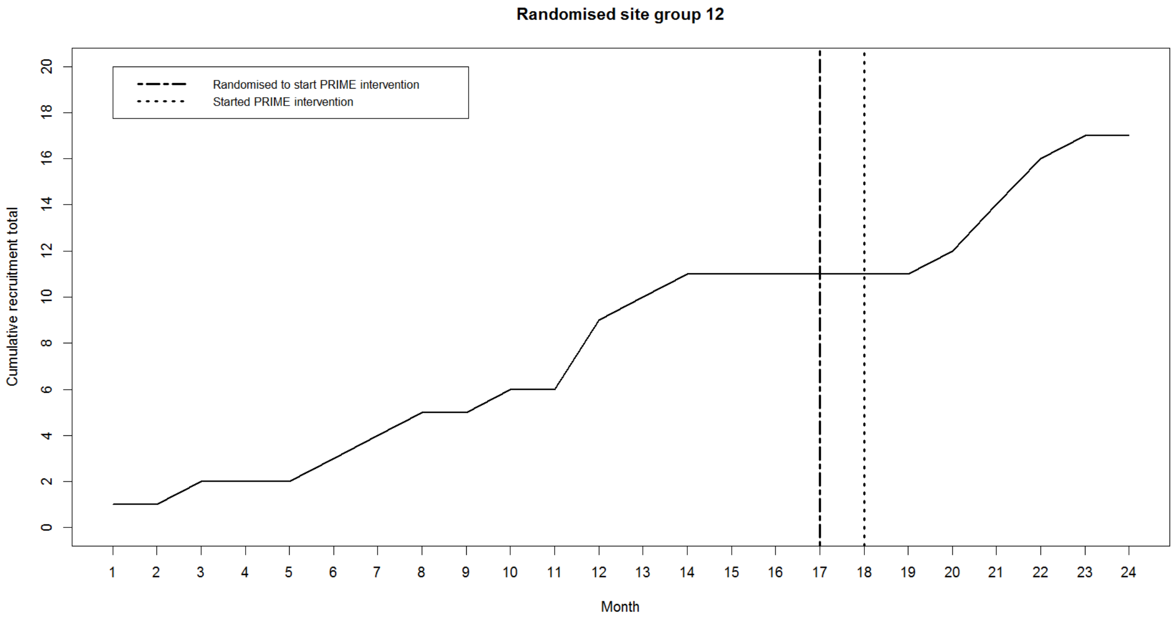

Supplement: Supplementary file 2 — Separate plots of each of the randomised groups of sites. (PDF 421 kb) [file 13063_2017_2355_MOESM2_ESM.pdf]
