# Supplementary material for: Promoting Recruitment using Information Management Efficiently (PRIME): a stepped-wedge, cluster randomised trial of a complex recruitment intervention embedded within the REstart or Stop Antithrombotics Randomised Trial
Source: Trials. 2017 Dec 28;18:623. doi: 10.1186/s13063-017-2355-z (PMC5745698; doi:10.1186/s13063-017-2355-z)
Supplement: Supplementary file 3 — Sensitivity analyses of the primary outcome. (PDF 278 kb) [file 13063_2017_2355_MOESM3_ESM.pdf]

**Appendix 3: Sensitivity Analyses on the primary outcome**

**Table S1** Negative-binomial GLMM results for an analysis of the primary outcome assuming an intervention-by-time interaction effect (N=1728, 72 sites)

| Variable | Rate Ratio | 95% Confidence Interval | P-value |
|---|---|---|---|
| Intervention (Reference category: Control condition) | 1.04 | 0.54 to 2.00 | 0.901 |
| Time since start of study | 1.01 | 0.96 to 1.07 | 0.630 |
| December/January (Reference: any other month) | 0.54 | 0.284 to 1.03 | 0.061 |
| Site location in Scotland (Reference: England/Wales) | 2.18 | 1.06 to 4.5 | 0.034 |
| Time since introduction of Recruitment Review | | | |
| 3-6 months | 0.92 | 0.46 to 1.81 | 0.799 |
| Over 6 months | 0.392 | 0.183 to 0.84 | 0.016 |
| (Reference 0-3 months) | | | |

**Table S2** Negative-binomial GLMM results for the **per-protocol analysis** of the primary outcome (using actual intervention timings) (N=1248, 52 sites)

| Variable | Rate Ratio | 95% Confidence Interval | P-value |
|---|---|---|---|
| Intervention (Reference category: Control condition) | 1.01 | 0.50 to 2.04 | 0.981 |
| Time since start of study | 0.97 | 0.92 to 1.02 | 0.288 |
| December/January (Reference: any other month) | 0.66 | 0.333 to 1.31 | 0.233 |
| Site location in Scotland (Reference: England/Wales) | 1.81 | 0.90 to 3.66 | 0.097 |

**Table S3** Negative-binomial GLMM results for the secondary analysis of the primary outcome excluding data from the pre- and post- rollout periods (N=792, 72 sites)

**Appendix 3: Sensitivity Analyses on the primary outcome**

| Variable | Rate Ratio | 95% Confidence Interval | P-value |
|---|---|---|---|
| Intervention (Reference category: Control condition) | 1.07 | 0.54 to 2.13 | 0.841 |
| Time since start of study | 0.94 | 0.86 to 1.04 | 0.246 |
| December/January (Reference: any other month) | 0.340 | 0.133 to 0.87 | 0.024 |
| Site location in Scotland (Reference: England/Wales) | 1.89 | 0.84 to 4.3 | 0.126 |
| Average number randomised in first 6 months | 18.2 | 2.00 to 166 | 0.010 |

**Table S4** Intervention effect results from Negative-binomial GLMMs of the primary outcome using different methods of specifying the time trend (N=1728, 72 sites)

| Method of specifying time trend | Intervention Effect Rate Ratio | Intervention Effect 95% Confidence Interval | P-value | AIC |
|---|---|---|---|---|
| Continuous linear term | 1.06 | 0.55 to 2.03 | 0.870 | 885.13 |
| Categorical time (categories are months) | 1.22 | 0.63 to 2.34 | 0.559 | 904.38 |
| Categorical time (categories in 5 monthly intervals) | 1.00 | 0.52 to 1.92 | 0.989 | 889.92 |
| Spline function: B-spline of order 2 with no interior knots | 1.06 | 0.58 to 1.92 | 0.858 | 885.13 |
| Spline function: B-spline of order 3 with no interior knots | 1.04 | 0.63 to 1.72 | 0.870 | 885.20 |
| Spline function: B-spline of order 3 with 3 interior knots | 1.21 | 0.62 to 2.33 | 0.578 | 889.22 |
| Spline function: B-spline of order 4 with 2 interior knots | 1.20 | 0.62 to 2.34 | 0.582 | 890.03 |
| Spline function: B-spline of order 4 with 3 interior knots | 1.21 | 0.63 to 2.33 | 0.574 | 890.16 |

AIC, Akaike Information Criterion
